# Wikipedia – challenges and new horizons in enhancing medical education

**DOI:** 10.1186/s12909-015-0309-2

**Published:** 2015-03-06

**Authors:** Verena G Herbert, Andreas Frings, Herwig Rehatschek, Gisbert Richard, Andreas Leithner

**Affiliations:** 1 Dermatologikum Hamburg, Stephansplatz 5, 20354 Hamburg, Germany; 2 Department of Ophthalmology, University Medical Centre Hamburg-Eppendorf, Hamburg, Germany; 3 Medical University of Graz, Graz, Austria; 4 Department of Orthopaedics, Medical University of Graz, Graz, Austria

**Keywords:** Wikipedia, Medical education, Professional responsibility, Evaluation of information

## Abstract

**Background:**

Wikipedia gains growing attention as a provider of health information. This study aimed to investigate the use, relevance and challenges of Wikipedia among medical students.

**Methods:**

An online questionnaire was made accessible to students at five medical universities in Germany, Austria, and Norway. Besides demographical data, the questions covered the role of Wikipedia in the academic life of medical students. The questionnaire investigated if the students had ever found erroneous medical entries and whether they corrected these.

**Results:**

A frequent use of Wikipedia in general is statistically significant correlated with a frequent use in medical studies (p < 0.001). Information retrieved from Wikipedia is predominantly critically appraised either by comparing it to profound knowledge (79%) and/or to specific literature (75%). Despite most (97%) respondents disclosed that they already had found false information in Wikipedia, recognized errors were seldomly corrected (~20%).

**Conclusions:**

The information retrieved from Wikipedia is critically appraised. However, we found shortcomings in handling erroneous entries. We argue for professional responsibility among medical students in dealing with this dynamic resource. Moreover, we encourage medical schools to supplement information to Wikipedia to further benefit from the vast possibilities of this platform.

## Background

At the beginning of 2012, the online encyclopaedia Wikipedia was blacked out as part of a societal protest against two U.S. anti-piracy bills that most people were hitherto unaware of [1,2]. Within days, seven million people had signed an online petition in effort to stop these bills [2]. The Internet, especially Wikipedia, had proven its importance in everyday life. Even the medical sector is influenced by Wikipedia’s omnipresence. It has gained considerable attention among both healthcare professionals and the lay public in providing medical information [3]. Patients rely on the information they obtain from Wikipedia before deciding to seek professional help [3,4]. As a result, physicians are confronted by a professional dilemma as patients weigh information provided by medical professionals against that on Wikipedia, the new provider of health information [5,6]. However, the online encyclopaedia is also commonly used by physicians from as early as medical school [7,8]. Despite evidence that Wikipedia is widely used among healthcare professionals, [9-11] there has been no detailed investigation evaluating its use in medical studies. This paper aims to show the role of Wikipedia in the academic life of medical students, thereby addressing the frequency of use, handling of erroneous entries and consequences for medical educators.

## Methods

Medical students from five universities (Medical University of Graz, Austria (MUG); Paracelsus Medical University, Salzburg, Austria (PMU); Charité Medical University Berlin, Germany (Charité); University Medical Centre Hamburg-Eppendorf, Germany (UKE); Medical Faculty of the Norwegian University of Science and Technology, Trondheim, Norway (NTNU)) at every stage of training were invited to participate in an online survey regarding Wikipedia’s role in their academic lives. All medical schools that were registered in Austria and Germany during 2013 were asked to join thisstudy, however, only the above mentioned universities gave their consent for participation. NTNU did participate as one of the authors (VH) was a student at this university. The voluntary survey ran between July and October 2014 and could be completed anonymously on a password secured website. The total questionnaire consisted of three demographic, 17 single- and one multiple-section questions. However, only questions related to the focus of the current paper are presented here (Table 1). Other questions were related to different topics (local medical libraries, non-digital medical literature, e-learning and buying behaviour related to medical literature; questions and data not published here).

**Table 1:** **Main questions with respect to the use of Wikipedia, the handling of retrieved information and dealing with erroneous information**

	Possible answers
**How often do you use wikipedia as an information source in general?**	
	Never
	1-5x/week
	Daily
	1-5x/month
**How often do you use Wikipedia as an information source in your medical studies?**	
	Never
	1-5x/week
	Daily
	1-5x/month
**Given that you read a medical article on Wikipedia, do you draw any criteria to evaluate its quality? (Multiple answer possible)**	
	I compare it to my knowledge
	Number of references
	Authors are well-known
	High number of revisions
	Date of latest revision
	I compare it to specific literature
	I discuss the content with my colleagues/teachers
	I do not draw any criteria
	I trust my gut feeling
**Given that you find incorrect information, what do you do?**	
	I correct the error
	I am not familiar how to correct articles
	I know how to correct articles but leave the entry unaltered
	I draw attention to the false information in any other way

The study protocol was approved by the local ethics committee (Medical University of Graz, EK-No. 24–002 ex 11/12). All completed questionnaires from medical students were included for evaluation and used to assess if

1. the student’s general use of Wikipedia is comparable to its use in medical studies,
2. the information retrieved from Wikipedia is critically appraised,
3. the participating students have ever found erroneous information in Wikipedia medical articles and have corrected the information.

Further, data has been itemized according to educational stage and gender. Using Pearson Chi-square test, the above-mentioned variables were examined for statistical significant results. To test for monotonicity across the groups, the Jonckheere-Terpstra-Test was performed. Descriptive data were analyzed using SPSS 19.0 (SPSS Inc, Chicago, IL), considering p-values ≤0.05 as statistically significant.

## Results

### Demographic data

Of approximately 7000 possibly reached students, 1442 (21%) returned questionnaires. Thereof, 1365 (20%) questionnaires were completely answered and therefore used for analysis. Fifty-three percent (n = 723) of the participants were female and 47% (n = 642) were male. The majority of 901 (66%) students was between 20 to 25 years old, 300 (22%) were aged below 20 and 164 (12%) were older than 25 years. At the time of the survey, 55% (n = 751) of the respondents were at pre-clinical and 45% (n = 614) at clinical stage.

### Students’ general and medical use of Wikipedia

Regarding general information seeking behaviour, 819 (60%) participants disclosed a moderate use of Wikipedia. Of those, 342 (56%) students were at clinical stage, 394 (61%) were male. Using Wikipedia in a high manner for searching general information, 127 (20%) were at clinical stage, 150 (24%) were male. In 145 (24%) cases, the students were at clinical stage and 98 (15%) were male of those, that stated a low general use (Table 2).

**Table 2:** **Characteristics of study participants (n = 1365) with different levels of general use of Wikipedia**

Variable	Level of general use of Wikipedia			P value
	*low (n = 328 (24%))*	*moderate (n = 819 (60%))*	*high (n = 218 (16%))*	
**Sex**				
*Male (n = 642)*	98 (15%)	394 (61%)	150 (24%)	0.000
*Female (n = 723)*	230 (32%)	425 (59%)	68 (9%)	
**Age group**				
*<20 (n = 300)*	123 (41%)	90 (30%)	87 (29%)	0.109
*20-25 (n = 901)*	145 (16%)	687 (76%)	69 (8%)	
*>25 (n = 164)*	60 (37%)	42 (26%)	62 (37%)	
**Stage of study**				
*Pre-clinical (n = 751)*	183 (24%)	477 (64%)	91 (12%)	0.027
*Clinical (n = 614)*	145 (24%)	342 (56%)	127 (20%)	

With respect to searching for medical information, the majority of 55% (n = 751) reported a moderate use. Of those, 333 (44%) students were at clinical stage, 386 (51%) were male. Using Wikipedia in a high manner for medical information seeking (n = 158 (12%)), 87 (55%) persons were at clinical stage, 94 (59%) were male. Finally, 194 (43%) were at clinical stage and 162 (36%) were male of those that stated a low medical use (Table 3).

**Table 3:** **Characteristics of study participants (n = 1365) with different levels of medical use of Wikipedia**

Variable	Level of medical use of Wikipedia			P value
	*low (n = 456 (33%))*	*moderate (n = 751 (55%))*	*high (n = 158 (12%))*	
**Sex**				
*Male (n = 642)*	162 (25%)	386 (60%)	94 (15%)	0.000
*Female (n = 723)*	294 (41%)	365 (50%)	64 (9%)	
**Age group**				
*<20 (n = 300)*	166 (55%)	81 (27%)	53 (18%)	0.063
*20-25 (n = 901)*	187 (21%)	614 (68%)	100 (11%)	
*>25 (n = 164)*	103 (63%)	56 (34%)	5 (3%)	
**Stage of study**				
*Pre-clinical (n = 751)*	262 (35%)	418 (56%)	71 (9%)	0.024
*Clinical (n = 614)*	194 (32%)	333 (54%)	87 (14%)	

Pearson Chi-square test showed a statistically significant correlation between the use of Wikipedia in general and its use in medical studies (p < 0.001). The use of Wikipedia in medical studies was statistically significant correlated with the students’ study progress (p = 0.024) and gender (p < 0.001). There was no statistical significance between the use of Wikipedia in medical studies and the respondents’ age (p = 0.063).

Nonparametric analysis using Jonckheere-Terpstra-Test showed an increasing trend of Wikipedia use in general with an increasing use of it in medical studies (p < 0.001). There was likewise a positive trend between the frequency of Wikipedia use in general and the students’ study progress (p = 0.027). A negative trend could be noted between the general frequency of use and the respondents’ gender (p < 0.001).

### Critical appraisal of information retrieved from Wikipedia

For assessing information retrieved from Wikipedia, n = 1074 (79%) reported comparing data to their knowledge and/or to specific literature (n = 1028 (75%)) (Table 4). Thirty-five percent (n = 723) discussed the article content with their colleagues and/or professors, whereas 25% (n = 341) evaluated the article’s quality according to its number of references and 16% (n = 218) to the date of its latest revision. A minority stated to not draw any criteria for assessing data quality (n = 109 (8%)) and to only trust their gut feeling (n = 111 (8%)) (Table 4). Familiarity of the authors (n = 82 (6%)) and number of revisions (n = 55 (4%)) played a minor role in assessing an article’s quality. Multiple answers were possible for this question.

**Table 4:** **Cross-tabulation of quality assessment criteria of information retrieved from Wikipedia with participants’ characteristics (n = 1365) (multiple answers possible)**

Variable	Quality assessment criteria				P value
	*Comparison with one’s own knowledge (n = 1074(79%))*	*Comparison with scientific literature (n = 1028(75%))*	*Nothing at all (n = 109(8%))*	*One’s gut feeling (n = 111(8%))*	
**Sex**					
*Male (n = 642)*	509	479	59	53	0.347
*Female (n = 723)*	565	549	50	58	
**Age group**					
*<20 (n = 300)*	214	45	21	26	0.592
*20-25 (n = 901)*	717	836	21	24	
*>25 (n = 164)*	143	147	67	61	
**Stage of study**					
*Pre-clinical (n = 751)*	597	579	45	55	0.042
*Clinical (n = 614)*	477	449	64	56	

Pearson Chi-square test showed a statistically significant correlation between appraising information critically and the participants study progress (p = 0.042). There was no statistical significance between reviewing information critically and age (p = 0.592), or gender (p = 0.347).

Nonparametric analysis using the Jonckheere-Terpstra-Test showed an increasing trend between frequency of Wikipedia use and study progress (p = 0.042).

### Handling erroneous Wikipedia articles

Asked if they have ever found inaccurate medical entries on Wikipedia, 1324 (97%) students affirmed this. Of those, 861 (65%) students did not know how to revise articles and 199 (15%) let the false information unaltered, despite knowing how to correct articles. In contrast, 159 (12%) corrected errors immediately and 66 (5%) of the respondents drew attention to the inaccurate information in any other way. Regarding the students that do not revise respectively do not know how to revise articles, 55% were in clinics and 51% were male. In concerns of correcting erroneous entries, 44% were in clinics and 47% were male (Table 4).

Pearson Chi-square test showed a statistically significant correlation between handling inaccurate information and gender (p < 0.001), use of Wikipedia in general (p < 0.001), and in medical studies (p < 0.001). There was no statistical significance related to age (p = 0.680) or study progress (p = 0.334).

Nonparametric analysis using the Jonckheere-Terpstra-Test showed a positive trend between handling false entries and gender (p < 0.001). A negative trend was noted between frequency of use in general and correcting erroneous entries (p < 0.001). There was also a statistically significant trend between frequency of use in medical studies and the correction of false information (p < 0.001).

## Discussion

### Information seeking and sharing behaviour

Wikipedia is an open web-based encyclopaedia and is therefore more responsive to changes in knowledge than conventional encyclopaedias. Without a doubt, Wikipedia is one of the most dominant online reference sources, gaining in presence, quality, and content [3] - also for retrieving health information [10,12,13]. Not only the lay public but health professionals, researchers, and medical students depend on it as a resource for medical information [7]. Studies show that 50–70% practicing and 70% junior physicians use Wikipedia as a medical information source [7]. In medical education, Wikipedia’s potential role is that of a vast learning resource. As we could demonstrate, the site is used by the majority of the surveyed students in context of their academic education. A strong correlation between searching for general and medical entries could be disclosed. However, its use by the majority does not necessarily confirm its reliability. The medical use of Wikipedia seems to increase in frequency with the respondents’ study progress. It could serve well to enhance the students’ information seeking and sharing behaviour in correlation to the stage of their medical education.

### Critical appraisal of information

Information literacy, [14,15] the ability to search, retrieve, and evaluate data according to quality, credibility and applicability, is crucial for academic success. Hence, serious concerns have been expressed regarding Wikipedia’s accuracy and value as an educational resource in medical studies [15,16]. As a result, users of Wikipedia must continually balance the need for easy to find and available with questionable information. Percentages obtained in this study show that information retrieved from Wikipedia is critically appraised by most of the participating students, correlating with their study progress. Interestingly, the majority state that they compare the information to specific literature and knowledge presented in their academic studies. Only a small number of students question the articles’ quality not at all or rely on their instincts. We argue that a certain progress in studies and the use of Wikipedia combined with reliable references, transforms information-seeking and -recording to a multifactorial, evolving process of highest value for the students and their academic development.

### Challenges in handling erroneous entries

The authors of Wikipedia articles usually remain anonymous and contributions can easily be changed, regardless of professional expertise [16]. However, Wikipedia is not only characterized by the factors “anytime, anywhere, and anybody”, but also by a constant review process, the “perpetual beta”, and the so-called “architecture of participation” [12]. This collective intelligence compels a “survival of the fittest” environment, whereby unsustainable articles should be revised. The vast majority of the interviewed students have found inaccurate medical entries on Wikipedia. However, recognized errors were corrected only by a minority. Users that access Wikipedia in a high manner tend to correct false information less often than those that use Wikipedia less frequently. This suggests that a higher degree of responsibility promotes selective use of Wikipedia for a more elaborate and reflected information search. 4.Limitations.

We acknowledge certain limitations to our study. There was a low response rate (about 20%) and the possibility of social desirability bias when responding to the survey could influence the obtained results. Moreover, the questionnaire was created without external review board.

## Conclusions

We state that Wikipedia should not be viewed as being inappropriate for its use in medical education. Given Wikipedia’s central role in medical education as reported in our survey, its integration could yield new opportunities in undergraduate education. High-quality medical education and sustainability necessitates the need to know how to search and retrieve unbiased, comprehensive, and reliable information. Students should therefore be advised in reflected information search and encouraged to contribute to the “perpetual beta” improving Wikipedia’s reliability. Therefore, we ask for inclusion in medical curricula, since guiding students’ use and evaluation of information resources is an important role of higher education. It is of utmost importance to establish information literacy, evidence-based practices, and lifelong learning habits among future physicians early on, hereby contributing to medical education of the highest quality.

Accordingly, this is an appeal to see Wikipedia as what it is: an educational opportunity. This is an appeal to academic educators for supplementing Wikipedia entries with credible information from the scientific literature. They also should teach their protégés to obtain and critically evaluate information as well as to supplement or correct entries. Finally, this is an appeal to medical students to develop professional responsibility while working with this dynamic resource. Criticism should be maintained and caution exercised since every user relies on the accuracy, conscientiousness, and objectivity of the contributor.
